# Rhamnolipids as Green Stabilizers of nZVI and Application in the Removal of Nitrate From Simulated Groundwater

**DOI:** 10.3389/fbioe.2022.794460

**Published:** 2022-04-19

**Authors:** Cinthia Cristine Moura, Ana Maria Salazar-Bryam, Rodolfo Debone Piazza, Caio Carvalho dos Santos, Miguel Jafelicci, Rodrigo Fernando Costa Marques, Jonas Contiero

**Affiliations:** ^1^ Associate Laboratory of the Institute for Research in Bioenergy (IPBEN)-Unesp, São Paulo State University (Unesp), Institute for Research in Bioenergy, Rio Claro, Brazil; ^2^ Industrial Microbiology Laboratory, General and Applied Biology, Institute of Biosciences, São Paulo State University (Unesp), Rio Claro, Brazil; ^3^ Laboratory of Magnetic Materials and Colloids, Institute of Chemistry, São Paulo State University (Unesp), Araraquara, Brazil; ^4^ Monitoring and Research Center for the Quality of Fuels, Biofuels, Petroleum and Derivatives (CEMPEQC/IQ-Unesp), Araraquara, Brazil

**Keywords:** rhamnolipids, stabilizer, NZVI, nitrate removal, groundwater

## Abstract

Environmental contamination caused by inorganic compounds is a major problem affecting soils and surface water. Most remediation techniques are costly and generally lead to incomplete removal and production of secondary waste. Nanotechnology, in this scenario with the zero-valent iron nanoparticle, represents a new generation of environmental remediation technologies. It is non-toxic, abundant, cheap, easy to produce, and its production process is simple. However, in order to decrease the aggregation tendency, the zero-iron nanoparticle is frequently coated with chemical surfactants synthesized from petrochemical sources, which are persistent or partially biodegradable. Biosurfactants (rhamnolipids), extracellular compounds produced by microorganisms from hydrophilic and hydrophobic substrates can replace synthetic surfactants. This study investigated the efficiency of a rhamnolipid biosurfactant on the aggregation of nanoscale zer-valent iron (nZVI) and its efficiency in reducing nitrate in simulated groundwater at pH 4.0. Two methods were tested: 1) adding the rhamnolipid during chemical synthesis and 2) adding the rhamnolipid after chemical synthesis of nZVI. Scanning electron microscopy field emission, X-ray diffractometry, Fourier transform infrared spectroscopy, thermogravimetric analysis, Dynamic Light Scattering, and zeta potential measurements were used to characterize bare nZVI and rhamnolipid-coated nZVI. The effects of the type of nZVI and initial NO_3_ concentration were examined. Nanoscale zer-valent iron with the addition of the rhamnolipid after synthesis achieved the best removal rate of nitrate (about 78%), with an initial nitrate concentration of 25 mg L^−1^. The results suggest that nZVI functionalized with rhamnolipids is a promising strategy for the *in situ* remediations of groundwater contaminated by NO_3_, heavy metal, and inorganic carbon.

## Introduction

Due to the growing demand for food and the great use of fertilizers to obtain it, the contamination of the soil and the aquifers has demanded studies to overcome the problem. Fertilizers has caused environmental pollution with threats to agricultural productivity, food security, ecosystem health, human health, and economic prosperity (Erisman et al., 2008; Klove et al., 2011; Zhang et al., 2015; Hansen et al., 2017; Velis et al., 2017). The World Health Organization and US Environmental Protection Agency have established the maximum contaminant level for nitrate (NO_3_) of 10 mg L^−1^ in drinking water (WHO, International Program On Chemical Safety, 1996; EPA, 2002).

In recent years, nanoparticles have been used in environmental remediation because of their great reduction power and surface area. Nanoparticle zero-valent iron (nZVI) has been extensively implemented for groundwater remediation due to its low cost and the ability to reduce oxidized pollutants (Crane and Scott, 2012; Yirsaw et al., 2015; Stefaniuk et al., 2016; Magthalin et al., 2016). However, iron nanoparticles have some drawbacks that need to be solved such as the issue of cluster formation due to their interfacial energy and high surface reactivity (Zhang, 2003; Zhao et al., 2016), as well as their instability, which can be easily oxidized by water or oxygen in their environment, forming a passive layer on their surface. To diminish the tendency toward aggregation, nZVI is often coated with surfactants (NSET, 2003; Crane and Scott, 2012), which play major roles in improving particle mobility (Dutra, 2015), lowering interfacial tension, and preventing the coalescence of newly formed drops (Morsy, 2014). Xue et al. (2018) investigated the performance of nanoscale nZVI coated with rhamnolipids (RL) in the immobilization of cadmium and lead. They demonstrated the effectiveness of nZVI in transforming labile cadmium and lead into a stable fraction, with an increase of 56.40 and 43.10% in the maximum residual percentage of these metals after 42 days of incubation, decreasing the mobility of the metals. Nitrate may also be chemically reduced by nZVI (Huang et al., 2011). The efficiency of nitrate removal by Fe^0^ is dependent on the pH value, with rapid reduction generally occurring at pH 2–4.5 (Hausmann and Syldatk, 1998; Choe et al., 2004; Huang and Zhang, 2004). nZVI is highly reactive in water, making it an excellent electron acceptor (Sturm and Morgan, 1996). Sharma et al. (2020) explored Fe_3_O_4_ nanoparticles prepared with a coating of rhamnolipids. These materials show to be monodispersed and stable in water under environmentally relevant pH and ionic strength values. These nanoparticles were used to remove dissolved inorganic carbons from water and showed high sorption capacity at pH 6 and pH 8 in both carbonate-free and in equilibrium with the atmosphere CO_2_ systems. The nanoparticles are non-toxic, abundant, inexpensive, and easy to produce (Zhang, 2003; Cook, 2009; Keane, 2010). The core consists primarily of zero-valent iron, whereas the mixed-valent oxide shell is formed as a result of the oxidation of metallic iron (Cornell and Schwertmann, 2004; Li et al., 2006; Crane and Scott, 2014). With rapid advances in biotechnology and increased environmental awareness, synthetic surfactants are increasingly being replaced with biologically produced compounds (Banat et al., 2000; Gautam and Tyagi, 2006). The aim of green synthesis and the stabilization of metallic nanomaterial is to decrease the use of chemical methods (Park et al., 2011). Microbial biosurfactants are extracellular compounds produced by microorganisms, such as bacteria, yeast, and filamentous fungi, grown on hydrophobic/hydrophilic carbon sources (Nitschke et al., 2005; Pirôllo et al., 2008; Tese, 2011; Lovaglio et al., 2015). Biosurfactants are surface-active molecules with both hydrophilic and hydrophobic moieties, which enable these compounds to interact at interfaces and reduce the surface tension of the medium (Mishra et al., 2009; Fernandes, 2011; Tese, 2011). Rhamnolipids used in this study are produced by the bacteria *Pseudomonas aeruginosa* that have been intensively investigated and extensively reviewed for various applications (Ochsner et al., 1996; Maier and Soberón-Chávez, 2000; Nitschke et al., 2005; Akiyode and Boateng, 2018; Moutinho et al., 2021; Varjani et al., 2021; Magri and Abdel-Mawgoud, 2022). Rhamnolipids are comprised of one or two molecules of rhamnose linked to one or two molecules of β-hydroxydecanoic acid (Desai and Banat, 1997; Hausmann and Syldatk, 1998; Banat et al., 2000). Rhamnolipids are promising candidates for the stabilization of nanoparticles. These natural compounds have been used to stabilize silver nanoparticles (Xie et al., 2006; Reddy et al., 2009; Kiran et al., 2010; Kumar et al., 2010; Ravi Kumar et al., 2010; Farias et al., 2014; Salazar-Bryam et al., 2021), nickel oxide (Palanisamy, 2008; Palanisamy and Raichur, 2009), cadmium sulfide (Singhal et al., 2010), iron oxide (Liao et al., 2010) and palladium-doped nanoscale zer-valent iron particles (Basnet et al., 2013; Bhattacharjee et al., 2016). To the best of our knowledge, there are no reports in the literature of rhamnolipid-mediated synthesis, stabilization, and application of nZVI for nitrate reduction. Therefore, the aim of the present study was to report evidence for the use of a low-cost rhamnolipid biosurfactant for the stabilization of nZVI in an aqueous solution and its use for the removal of nitrate from simulated groundwater.

## Materials and Methods

### Materials

Ferric chloride (FeCl_3_ 6H_2_O, 98%), Sodium borohydride (NaBH_4_, 97%), and Potassium bromide (KBr, 99%) were purchased from Sigma-Aldrich Chemical Corporation Sodium hydroxide (NaOH, 97%), ammonium chloride (NH_4_Cl, 99%), and sodium nitrate (NaNO_3_, 99%) were purchased from Labsynth and phosphoric acid (H_3_PO_4_, 95%) was purchased from J. T. Baker, Acetone (Honeyell, 100%) and deionized water were used for nZVI synthesis. For rhamnolipid production a Ca-free mineral salt medium containing glycerol as a carbon source was used (Müller et al., 2011). All of the components are of analytical grade and used without further purification. Further de-ionized water was used in all the experiments.

### Production and Extraction of Rhamnolipids

The strain *Pseudomonas aeruginosa* LBI 2A1, was obtained in previous work as a part of a doctoral thesis of Lovagio (Lovaglio, 2011). It was maintained in Lysogenic Broth (LB) plus 20% glycerol at −20°C. For pre-culture, the microorganism was inoculated into 25 ml of LB, then transferred to 200 ml of LB medium containing phosphate buffer solution (pH 6.8) and 1% (w/v) of glycerin. The system was kept on a rotary shaker for 48 h at 180 rpm and 32°C. The pre-inoculum culture (10% v/v) at an optical density of 0.08 (OD580) was transferred to 400 ml of production medium containing glycerol 2% (w/v). The pH of the medium was adjusted to 7 by adding NaOH 1 mol L^−1^. The culture was incubated on a rotatory shaker for 120 h at 200 rpm and 32°C. Cells were separated by centrifugation at 12,000 rpm for 30 min at 4°C, and the cell pellet was discarded. To the supernatant was added 85% H_3_PO_4_ 1:100 (v/v) to adjust of pH of about 2-3 and ethyl acetate 1:1,25 (v/v) for extraction of rhamnolipids. The mixture was shaken for 15 min and allowed to settle down until the phase separation. The inorganic phase was removed and the operation was repeated once again with the organic phase and ethyl acetate 1:1,25 for total extraction of rhamnolipids. After that, the organic phase containing the biosurfactant was concentrated using a rotary evaporator.

### Surface Activity Measurements and Structural Characterization of Rhamnolipids

Surface tension was determined by the Du-Noüy ring method with a Krüss K6 Tensiometer (Krüss, Hamburg, Germany). Ultrapure water was measured to calibrate the tensiometer. Experiments were performed at room temperature; all measurements were made in triplicate.

### Synthesis and Green Stabilization of nZVI

The synthesis was based on the borohydride reduction method (Wang and Zhang, 1997. The synthesis of nZVI was conducted in a beaker by adding sodium borohydride (6 mmol) dissolved in 2 ml of purified water to 40 ml ferric iron solution (4 mmol), for the reduction of ferric iron to nZVI. The solution was vigorously stirred with a magnetic bar at room temperature. The entire process was carried out in an argon atmosphere. The reduction reaction is as follows (Wang and Zhang, 1997):

$$\mathrm{Fe}{\left(H_{2}O\right)}_{6}^{3+}+{3BH}_{4}^{-}+3H_{2}O\to Fe^{0}+3B{\left(\mathrm{OH}\right)}_{3}+\mathrm{10}\mathrm{.5}H_{2}$$ (1)

A black precipitate formed instantly. After 20 min of reaction, the solid was magnetically decanted and washed three times with acetone.

Two stabilization methodologies of nZVI (Table 1) were compared with the bare-nZVI: synthesis of nZVI with rhamnolipids addition in ferric chloride solution (nZVI-A) and nZVI stocked in rhamnolipids solution (n-ZVI-S). The rhamnolipid solution used for nZVI-A and nZVI-S was above 250 mg L^−1^ (CMC, critical micellar concentration). The separation of the generated iron particles was achieved with a magnet, followed by washing with acetone at least three times.

**TABLE 1:** nZVI stabilization method.

Sample	RL
bare-nZVI	Without rhamnolipids
nZVI-A	Rhamnolipids addition in ferric chloride solution
nZVI-S	Stocked in rhamnolipids solution

### Nanoparticle Characterization

Characterization of the crystalline phase was determined by X-ray powder diffraction (XRD) using a Simiens D5005 diffractometer with a Cu Kα radiation source. The samples were recorded at 5–80° of 2θ, with a step of 0.02°. The Crystallographic Search-Match software was used to index the samples. Fourier transform infrared (FTIR) spectroscopy was used to confirm the obtainment of a rhamnolipid. Thermogravimetric analysis (TGA) was carried out to determine the total amount of nZVI and mass of the rhamnolipids, using the STA 409C/CD system from NETZSCH Instruments. Samples (15 mg) were analyzed from room temperature up to 700°C under 50 ml min^−1^ airflow with a heating rate of 10°C.min^−1^. The shape and morphology of the dried nanoparticles were determined using scanning electron microscopy (SEM) model JEOL 7500F, with an acceleration of 2kV. The hydrodynamic diameter and zeta potential measurements of the particles were evaluated by Dynamic Light Scattering (DLS) and electrophoretic mobility using laser Doppler electrophoresis in a Zeta Sizer NanoZS from Malvern Instruments.

### Nitrate Reduction Tests

Batch experiments were conducted using 1 L bottles at room temperature under light-excluding conditions. To create an anaerobic environment, the deionized water used for the preparation of the nitrate solution was boiled and the bottles were then purged with nitrogen gas to remove dissolved oxygen. Each bottle was filled with 1 L of initial NaNO_3_ concentrations (C0) of about 25, 50, and 100 mg L^−1^ NO_3_. At the onset of the experiment, the pH was adjusted to 4 using HCl 1 mol L^−1^ and 5 g of nZVI-S, nZVI-A, or bare nZVI were added to each bottle. Control experiments without the addition of nZVI nanoparticles were carried out in parallel. Samples (10 ml) were withdrawn every 15 min for 2 h. All experiments were performed in triplicate. The removal efficiency was calculated according to Eq. 2

$$removal\;efficiency\;\%=\frac{\left(C_{0}-C_{f}\right)}{C_{0}}\times 100$$ (2)

in which C0 is the initial NO_3_ concentration and Cf is the final NO_3_ concentration. Nitrate and ammonium were quantified with a Thermo Scientific™ Orion™ nitrate electrode and ammonia electrode, respectively. A kinetic model for nitrate reduction by nZVI can be described by pseudo-first-^order reaction kinetics^ (kobs). According to this model, the reaction rate is proportional to the nitrate concentration, as given in the following Eq. 3:

$$ln\left(\frac{C}{C_{0}}\right)=-k_{obs}$$ (3)

in which C_0_ is the initial NO_3_ concentration and C is the NO_3_ concentration at time t.

## Results and Discussion

### Rhamnolipid Production

**FIGURE 1:**
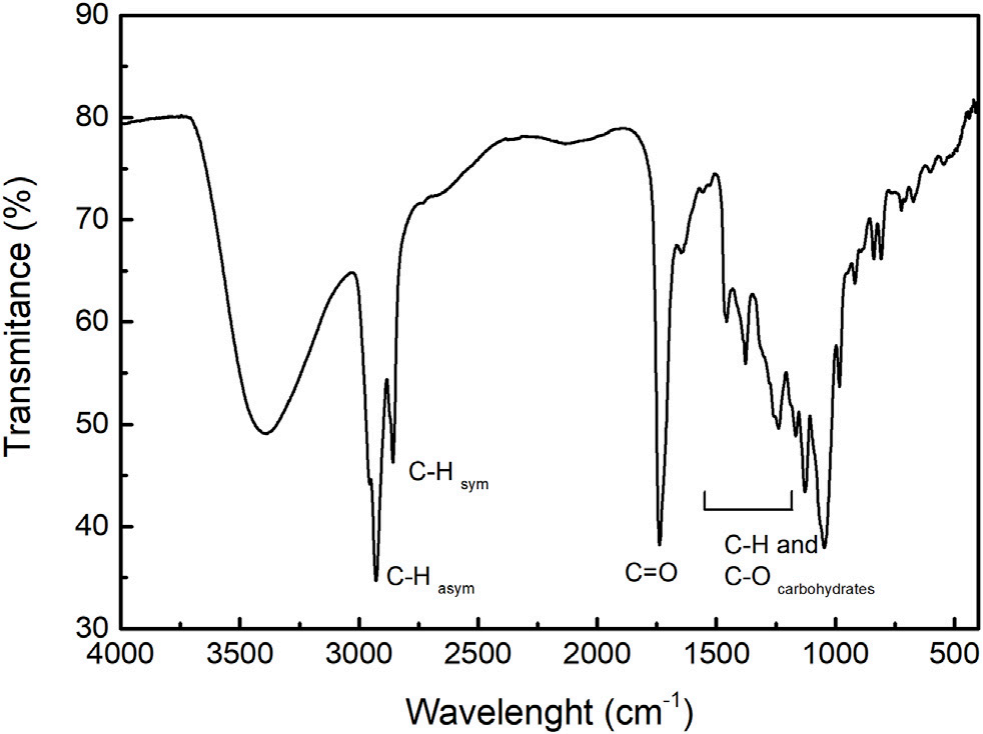
FTIR spectrum of rhamnolipids.

The biosurfactant from *P. aeruginosa* LBI 2A1 was cultivated in a low-cost medium and formulated using an agro-industrial substrate based on 2% (w/v) glycerol as a carbon source. The biosurfactant was produced for 120 h at 32°C. The CMC of the crude biosurfactant was evaluated by the Du-Noüy ring method and its value was determined at 250 mg L^−1.^ Although other authors have obtained 40.7 g/L, using soybean oil and ammonium nitrate (Sun et al., 2021), the use of a hydrophobic carbon source is observed, which for the strain used in this article, we have already reached up to 70.9 g/L using sunflower oil as carbon source (data not shown). This work concern to use of a by-product of the Biodiesel industry, such as glycerol. The FTIR analysis of the pure rhamnolipid is shown in Figure 1. The double bands at 2,922 and 2,854 cm^−1^ are assigned to C-H stretching vibrations of aliphatic groups. The band at 1,735 cm^−1^ corresponds to C=O stretching bonds of ester and carboxylic acid groups. The bands between 1,230 and 1,450 cm^−1^ are typical of C-H and O-H vibrations of carbohydrates, i.e., rhamnose units (Leitermann et al., 2008).

### Characterization of nZVI

**FIGURE 2:**
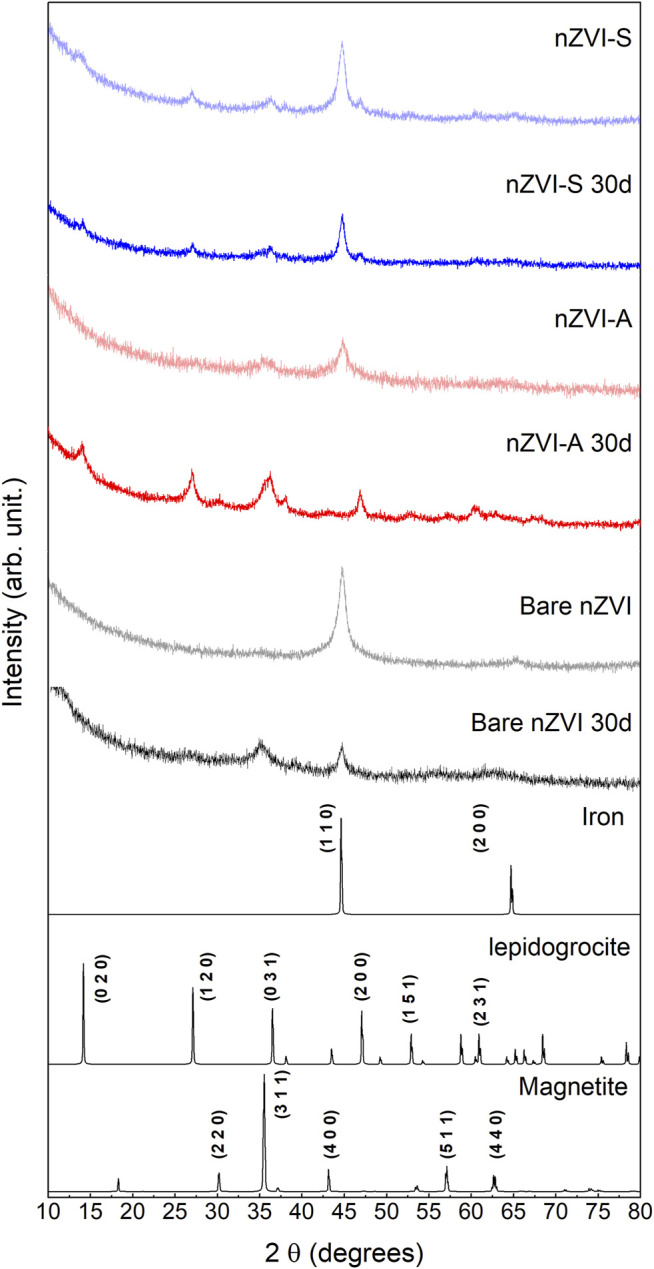
X-ray diffraction peaks associated with nZVI particles were recorded: freshly synthesized samples (lighter colour) and 30-day old samples (darker colour). The Pattern Diffraction File (PDF) of metallic iron (PDF 87-7194), lepidocrocite (PDF 8-98) and, magnetite (PDF 74-419) were displayed as well.

The diffractograms of the synthesized samples are shown in Figure 2. After synthesized, the samples were evaluated immediately and submitted to XRD analysis after 30 days. The bare nZVI sample was indexed as metallic iron (PDF 87-7194), the peaks of which correspond to (1 1 0) and (2 0 0) crystalline planes. After 30 days, it was observed the decreasing of the (1 1 0) crystalline iron plane and the arising of the (3 1 1) plane from the magnetite phase (PDF 74–419) indicates sample oxidation. The diffractogram of the nZVI-A sample revealed the presence of different crystalline phases. Besides metallic iron, magnetite (3 1 1) was also observed. Oxidation was also observed after 30 days, in which the characteristic peaks of metallic iron disappeared, and the sample was indexed as a mixture of magnetite and lepidocrocite (PDF 8–98) phases. On the contrary, the nZVI-S sample had better stabilization by rhamnolipids, and the metallic iron phase was present on freshly synthesized and 30-day old samples. Therefore, adding the metallic iron nanoparticles to the rhamnolipids solution results in a more effective surface functionalization protocol against oxidation. Scherrer’s equation was used to calculate the average crystallite diameter (D_XRD_). Table 2 corroborates the SEM data, demonstrating the nanometric size. The desired crystallinity phase was obtained. The estimated D_XRD_ of the nanoparticles shows different sizes among the methods used. The D_XRD_ increased as follows: nZVI-A< nZVI-S< nZVI.

**TABLE 2:** Average crystallite diameter according to Scherrer’s equation. * Crystallographic plane (1 1 0) and ** (3 1 1).

Sample	Crystallite diameter (nm)
nZVI-S	7.88 *
nZVI-S 30 d	7.16 *
nZVI-A	6.52 **
nZVI-A 30 d	4.77 *
nZVI	9.05 *
nZVI 30 d	11.3 **

**FIGURE 3:**
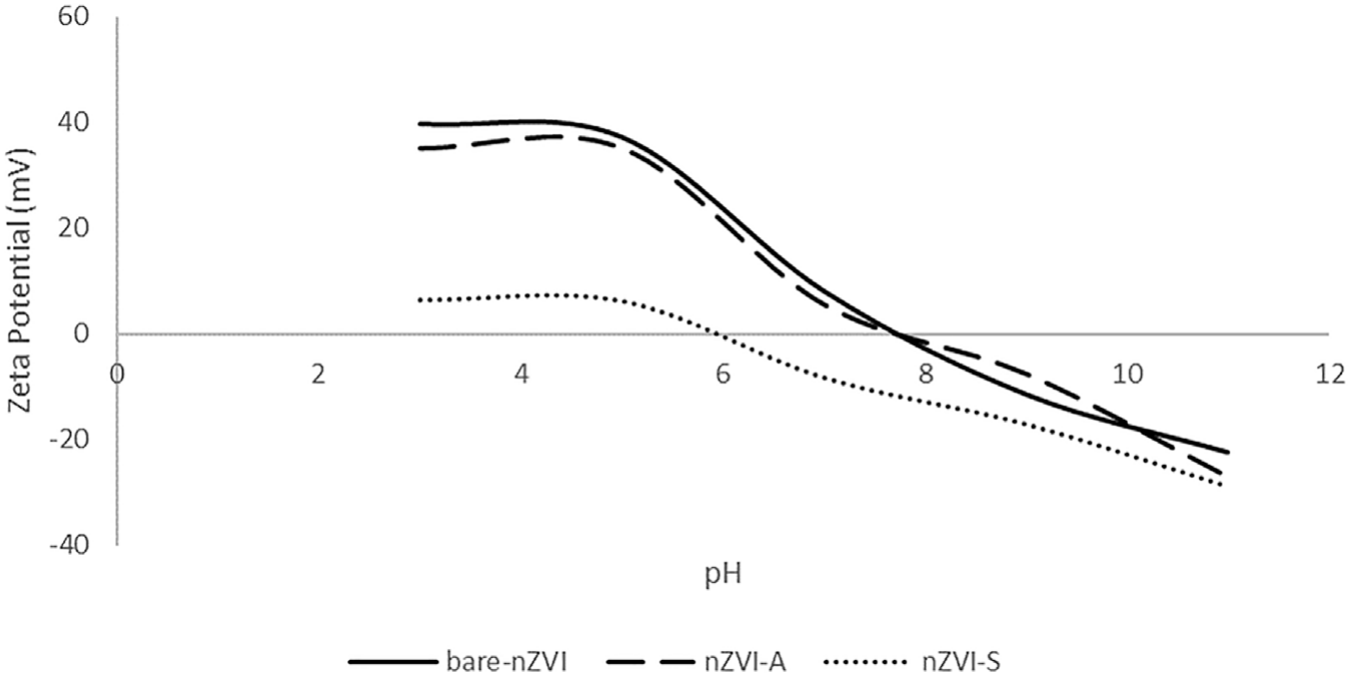
Zeta potential and pzc of nZVI.

The surface features of the nanoparticles were evaluated through zeta potential measurements. A zeta potential greater than ±30 mV indicates reasonably stable nanoparticles with low aggregation capacity due to charge equilibrium (Lowry et al., 2016; Hunter, 1988). Figure 3 shows different isoelectric points according to the method used to functionalize the surface nanoparticles. The bare nZVI and nZVI-A samples exhibited the same profile curve with an isoelectric point (point of zero charge) (pHpzc) at 7.8, which is compatible with the range of values found in the literature (7.5–8.9) (Su et al., 2011; Markova et al., 2014; Wen et al., 2014; Arancibia-Miranda et al., 2016; Habish et al., 2017). However, a shift was observed for nZVI-S, with a reduction in pHpzc to 6, which shows that the nZVI-S sample had more adequate coating when the iron nanoparticle was functionalized after synthesis due to the similarity between the rhamnolipid pKa and pHpzc values^.^ The pKa of the rhamnolipid is 5.6 (Sousa et al., 2014), while the observed pHpzc was 6, demonstrating that the surface corresponds to the rhamnolipid rather than the nanoparticles. Regarding colloidal stability, pH 4 was chosen to evaluate the zeta potential, once this is the point which materials will be applied. The results show that the bare nZVI sample had the highest value (37.2 mV), followed by nZVI-A (35.1 mV) and nZVI-S (6.3 mV). Although the nZVI-S sample had the lowest zeta potential value, the coating of the magnetic core by the rhamnolipids conferred steric stability to the compound.

**FIGURE 4:**
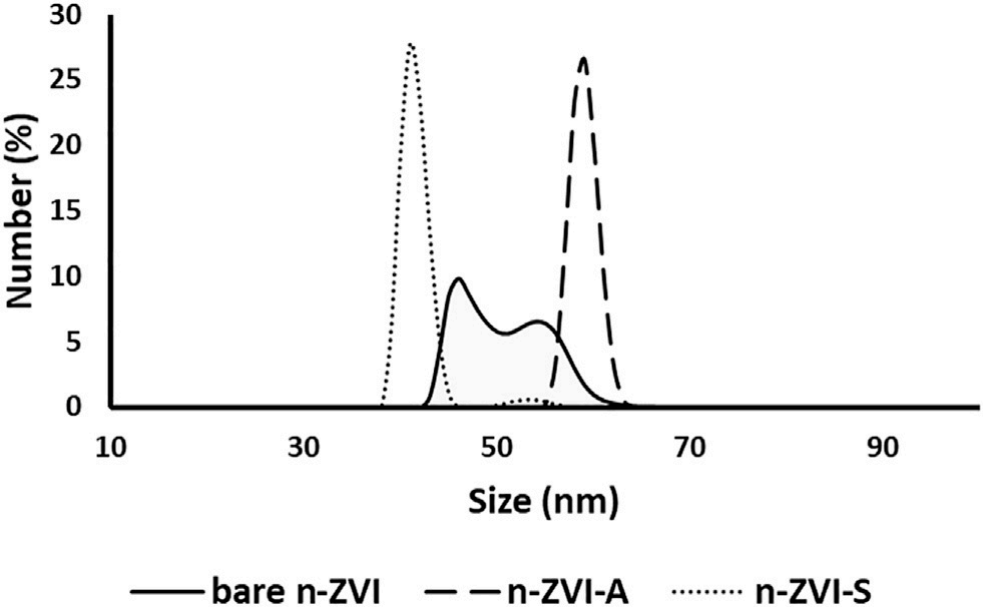
Average particle diameter size distribution of nZVI particles.

**FIGURE 5:**
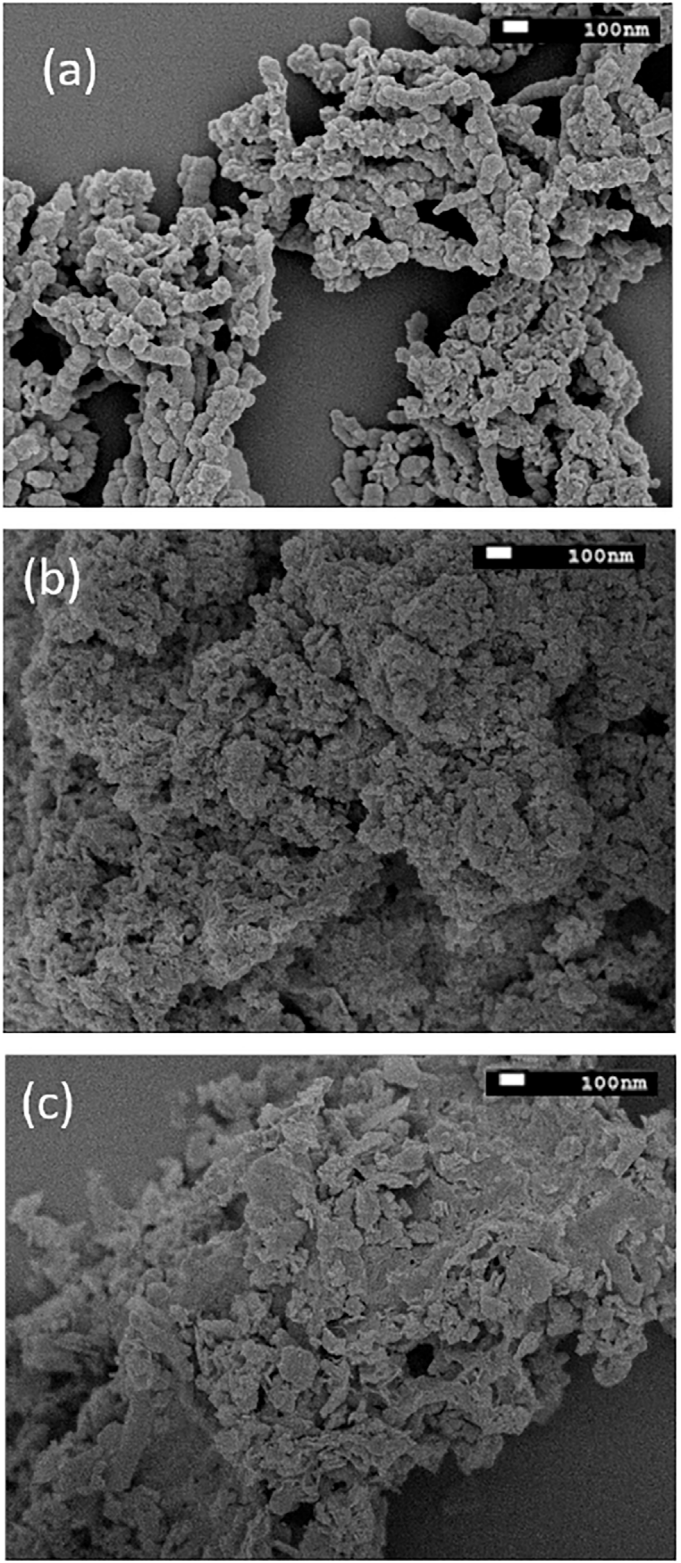
SEM images of **(A)** bare-nZVI **(B)** nZVI-A **(C)** nZVI-S.

The average diameter size of nZVI is shown in Figure 4. The bare nZVI had a shortened peak with a large base and an average diameter of 50 nm, while the average diameter of the nZVI-A and nZVI-S samples was approximately 60 and 42 nm, respectively. The larger average diameter for nZVI-A could be related to the addition of the rhamnolipid, as this biosurfactant has acid pH, which, in this situation, led to the formation of large aggregates, as observed by Dahrazma, et al. (2008) and Ishigami, et al. (1987). The formation of these aggregates is evidenced in the DLS analysis and SEM images (Figure 5).

Figure 5 displays the SEM images of bare nZVI, nZVI-A and nZVI-S. Bare nZVI was agglomerated in large clusters (Figure 5A). The morphology of nZVI differed depending on the time of the addition of rhamnolipids. As shown in Figure 5B, nZVI-S had less dispersed and smaller nanoparticles, whereas those of nZVI-A tended to be more agglomerated and consequently slightly larger (Figure 5C). This pattern was confirmed by the DLS analysis.

**FIGURE 6:**
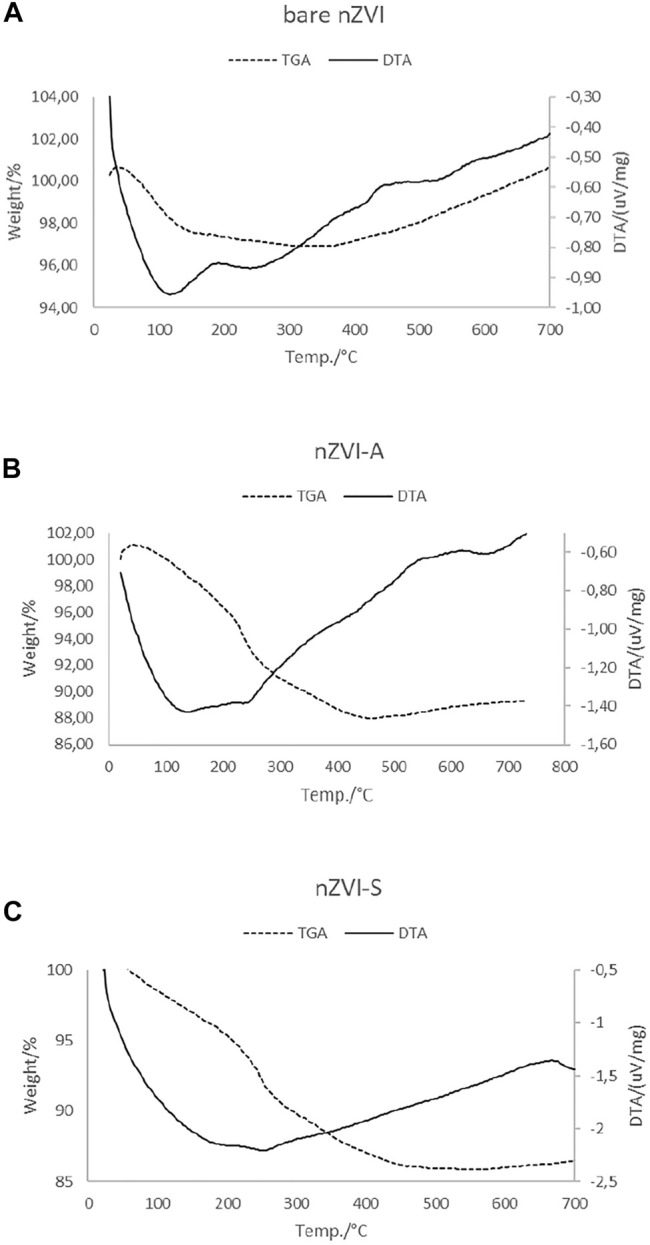
TGA and DTA curves for **(A)** bare-nZVI, **(B)** nZVI-A and **(C)** nZVI- S.

The thermal behavior of samples was investigated by TGA and DTG. The results are shown in Figure 6.

It is possible to identify two different regions in the TGA curve for bare-nZVI. The first region between 50 and 120°C, corresponds to a weight loss, due to the removal of physically bounded water and acetone from synthesis (Habish et al., 2017). The region near 189°C shows a slight change in the baseline without a peak and the DTA curve shows an exothermic peak, indicating the dehydration of the sample. In the second region, over 400°C, the gain mass is continuous at approximately 3%, the DTA curve presents an exothermic peak indicating oxidation on the bulk of the nanoparticle (Földvári, 2011). For the nZVI-A and nZVI-S samples, the TGA curve show three regions and the weight percentages are shown in Table 3.

**TABLE 3:** Regions of weight loss and gain (%) of nZVI-A and nZVI-S samples.

Region	Temperature °C	Weight	nZVI-A (%)	nZVI-S
I	50–230	Loss	8	9%
II	230–480	Loss	5	5%
III	480–700	Gain	1	0.5%

The first region of weight loss, comprising between 40 and 230°C, corresponds to the removal of residual water and acetone from synthesis (Habish et al., 2017). In this range, the DTA curve indicates an endothermic peak at 100°C, which corresponds to a dehydration reaction. The second region of weight loss occurs with a slight change in the baseline without a peak between 230 and 480°C, due to decomposition and elimination of organic backbone from rhamnolipids (Pui et al., 2013). The third region, over 480°C, is characterized by a slight gain of weight due to the oxidation of the nZVI. The DTA curve of nZVI-A and nZVI-S samples shows endothermic peaks. The DTA curve shows exothermic signals at 230 and 480°C which is in agreement with to weight loss phenomena. Over 480°C the DTA curve also show exothermic characteristic, that is associated with weight gain associated with the oxidation of the nZVI particles.

### Nitrate Reduction by nZVI Under Low pH Conditions

Fe^0^ is thermodynamically unstable in water. Dissolved oxygen is an oxidant and causes rapid corrosion of iron [Eq. 4]. On the other hand, under anaerobic conditions, water serves as the oxidant and corrosion takes place, producing hydrogen gas and hydroxide ions [Eq. 5]. The corrosion process results in an increase in pH media. Under acidic and anoxic conditions, the corrosion rate of iron is faster than iron corrosion by water [Eq. 6].

$$\mathrm{2F}e^{0}+O_{2}\mathrm{+2}H_{2}O\to 2Fe^{\mathrm{2+}}\mathrm{+4O}H^{-}$$ (4)

$$Fe^{0}\mathrm{+2}H_{2}O\to Fe^{\mathrm{2+}}+H_{2}\mathrm{2O}H^{-}$$ (5)

$$Fe^{0}\mathrm{+2}H^{+}\to Fe^{\mathrm{2+}}+H_{2}$$ (6)

Several pathways of nitrate reduction by nZVI have been proposed, such as the following equations.

$$\mathrm{4F}e^{0}\mathrm{+N}O_{3}^{-}\mathrm{+7}H_{2}O\to NH_{4}^{+}\mathrm{+4F}e^{\mathrm{2+}}\mathrm{+10O}H^{-}$$ (7)

$$\mathrm{5F}e^{0}\mathrm{+2N}O_{3}^{-}\mathrm{+6}H_{2}O\to N_{2}^{+}\mathrm{+5F}e^{\mathrm{2+}}\mathrm{+12O}H^{-}$$ (8)

**FIGURE 7:**
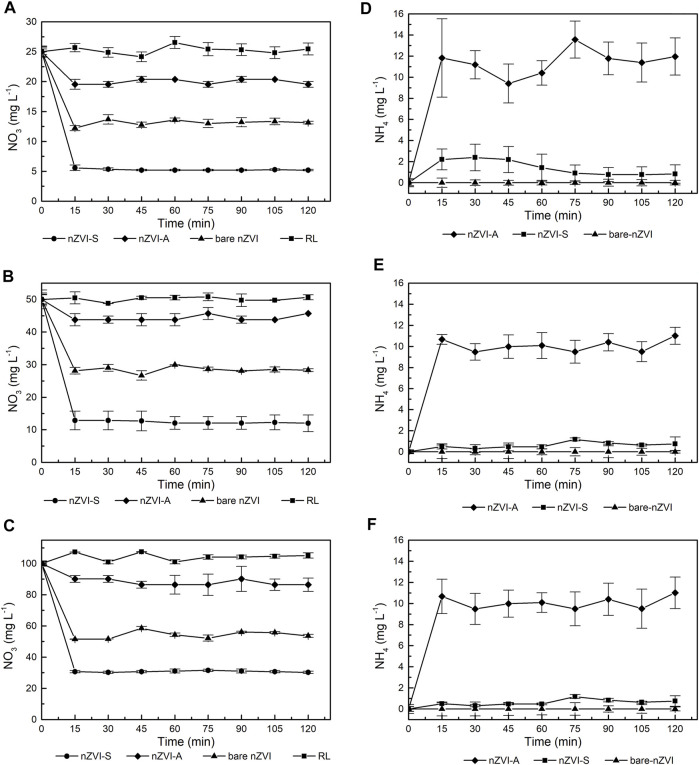
Effect of time and initial nitrates concentration on nitrates reduction using nZVI at pH 4 **(A)** 25 mg/L NO_3_ **(B)** 50 mg/L NO_3_ **(C)** 100 mg/L NO_3_ and effect of time and initial nitrates concentration on ammonia concentration using nZVI at pH 4 **(D)** 25 mg/L NO_3_ **(E)** 50 mg/L NO_3_ **(F)** 100 mg/L NO_3_.

Figure 7 compares the nitrate reduction rates achieved with bare nZVI, nZVI-S and, nZVI-A samples. The nitrate reduction was observed in the first 15 min; thereafter, the reaction remained nearly constant. The nZVI-S nanoparticles exhibited a higher kinetic constant than bare ZVI and nZVI-A, respectively, as shown in Table 4. In the first 15 min of the reaction, nZVI-S achieved nitrate efficiency removal of 78.62%, 77.65% and 68.89% for 25, 50, and 100 mg L^−1^ of NO_3_ solution, respectively. The nitrate removal with nZVI-A was comprised between 20.18 and 12.45%, while the bare nZVI were approximately 47, 57%, and 43.54%. The control group test shows no nitrate reduction in presence of the biosurfactant.

**TABLE 4:** Observed pseudo-first-order rate coefficient of nitrate reduction with nZVI.

NO_3_ (mg L^−1^)	Bare-nZVI		nZVI-S		nZVI-A	
	Efficiency removal (%)	k_obs_	Efficiency removal (%)	k_obs_	Efficiency removal (%)	k_obs_
25	47.57	0.043	78.62	0.107	20.18	0.015
50	43.54	0.038	77.65	0.101	12.45	0.009
100	46.29	0.041	68.89	0.078	13.62	0.010

To quantify the reaction rate, a pseudo-first-order reaction model (Eq. 3) was used to fit the kinetic data (Johnson et al., 1996). A high initial reduction rate was found when the initial concentration of nitrate was low. The observed surface reaction rate constant (kobs) (Table 4) for nZVI-S increased from 0.078 to 0.107 min^−1^ at a 15-min reaction time when the initial nitrate concentration was decreased from 100 to 25 mg L^−1^. For bare nZVI and nZVI-A, the kobs remained nearly constant, with values of 0.043 and 0.015, respectively, when the initial concentration was 25 mg L^−1^. The reaction did not slow down because of insufficient iron, since iron was added in stoichiometric excess in all experiments. The amount of nZVI (5 g) was not enough to react with the initial nitrate concentration according to the chemical reactions described above (Eqs 5, 6). Hausmann and Syldatk (1998), found complete nitrate reduction when the Fe^0^ concentration was increased to 10 g L^−1^.

Figures 7A–D demonstrates that the nitrate removal rate diminished substantially over time. Hydroxide ions were formed as nitrate was reduced. Since no additional acid was added, the OH^−^ accumulated, thus reducing the nitrate removal reaction (Hausmann and Syldatk, 1998; Liu et al., 2014). This behavior is consistent with the results presented in Figure 3, indicating that there was a surface modification on the nanoparticle. The concentration of ammonia did not increase with an increase in the initial concentration of nitrate (Figures 7D–F). Approximately 45% of nitrate was transformed into ammonia using 5 g of nZVI, showing that nZVI-S followed reaction Eqs 5, 6, whereas bare nZVI and nZVI-A did not transform nitrate into ammonia, probably following only Eq. 6.

## Conclusion

The present study presents the potential of using the rhamnolipid biosurfactant, obtained in a sustainable way (use of glycerol as a carbon source), in the preparation of nZVI as a stabilizer, increasing its stability and performance in terms of NO_3_ removal. Rhamnolipids are a promising alternative to synthetic surfactants for the synthesis and surface functionalization of zero valence iron nanoparticles. With the success achieved in this application, one more use of the rhamnolipid produced by *P. aeruginosa* LBI 2A1 is potentiated. The nanoparticles prepared with the rhamnolipid coating remained stable for 1 month, thus showing the efficiency of the process. The XRD analysis showed that the Fe^0^ intensity decreased gradually over time for both bare nZVI and nZVI-A, whereas a better response was found for nZVI-S, with the rhamnolipid concentration above the CMC. Compared to bare nZVI particles, nZVI-A and nZVI-S were stable in an aqueous solution and not easily oxidized and/or aggregated. Based on the efficient removal capacity and the observed first-order coefficient (kobs), NO_3_ removal by the different prepared materials followed the order of nZVI-S > bare nZVI > nZVI-A. In the presence of bare nZVI, most of the NO_3_ was not converted into NH_4_. For successful long-term groundwater field treatments using nZVI coated with rhamnolipids, a more detailed study of the chemical processing as well as the application of nanoparticles *in situ* should be carried out since the focus of this study was to verify the potential of nanoparticles obtained in the removal of nitrate from the aqueous medium. The results presented lead to the conclusion that the application of rhamnolipids as a coating for zero iron nanoparticles has potential and adds to those existing in the literature for use in the removal of metals such as cadmium and lead, since there is no mention of the use of rhamnolipids in the stabilization of zero iron nanoparticles to remove NO_3_ from groundwater.

## Data Availability

The original contributions presented in the study are included in the article/supplementary material, further inquiries can be directed to the corresponding author.
